# Oxidative Stress and Cardiovascular Risk in Type 1 Diabetes Mellitus: Insights From the DCCT/EDIC Study

**DOI:** 10.1161/JAHA.117.008368

**Published:** 2018-05-09

**Authors:** W.H. Wilson Tang, Paula McGee, John M. Lachin, Daniel Y. Li, Byron Hoogwerf, Stanley L. Hazen, DCCT/EDIC Research Group, D.M. Nathan, B. Zinman, O. Crofford, S. Genuth, J. Brown‐Friday, J. Crandall, H. Engel, S. Engel, H. Martinez, M. Phillips, M. Reid, H. Shamoon, J. Sheindlin, R. Gubitosi‐Klug, L. Mayer, S. Pendegast, H. Zegarra, D. Miller, L. Singerman, S. Smith‐Brewer, M. Novak, J. Quin, Saul Genuth, M. Palmert, E. Brown, J. McConnell, P. Pugsley, P. Crawford, W. Dahms, N.S. Gregory, M.E. Lackaye, S. Kiss, R. Chan, A. Orlin, M. Rubin, D. Brillon, V. Reppucci, T. Lee, M. Heinemann, S. Chang, B. Levy, L. Jovanovic, M. Richardson, B. Bosco, A. Dwoskin, R. Hanna, S. Barron, R. Campbell, A. Bhan, D. Kruger, J.K. Jones, P.A. Edwards, A. Bhan, J.D. Carey, E. Angus, A. Thomas, A. Galprin, M. McLellan, F. Whitehouse, R. Bergenstal, M. Johnson, K. Gunyou, L. Thomas, J. Laechelt, P. Hollander, M. Spencer, D. Kendall, R. Cuddihy, P. Callahan, S. List, J. Gott, N. Rude, B. Olson, M. Franz, G. Castle, R. Birk, J. Nelson, D. Freking, L. Gill, W. Mestrezat, D. Etzwiler, K. Morgan, L.P. Aiello, E. Golden, P. Arrigg, V. Asuquo, R. Beaser, L. Bestourous, J. Cavallerano, R. Cavicchi, O. Ganda, O. Hamdy, R. Kirby, T. Murtha, D Schlossman, S. Shah, G. Sharuk, P. Silva, P. Silver, M. Stockman, J. Sun, E. Weimann, H. Wolpert, L.M. Aiello, A. Jacobson, L. Rand, J. Rosenzwieg, D.M. Nathan, M.E. Larkin, M. Christofi, K. Folino, J. Godine, P. Lou, C. Stevens, E. Anderson, H. Bode, S. Brink, C. Cornish, D. Cros, L. Delahanty, . eManbey, C. Haggan, J. Lynch, C. McKitrick, D. Norman, D. Moore, M. Ong, C. Taylor, D. Zimbler, S. Crowell, S. Fritz, K. Hansen, C. Gauthier‐Kelly, F.J. Service, G. Ziegler, A. Barkmeier, L. Schmidt, B. French, R. Woodwick, R. Rizza, W.F. Schwenk, M. Haymond, J. Pach, J. Mortenson, B. Zimmerman, A. Lucas, R. Colligan, L. Luttrell, M. Lopes‐Virella, S. Caulder, C. Pittman, N. Patel, K. Lee, M. Nutaitis, J. Fernandes, K. Hermayer, S. Kwon, A Blevins, J. Parker, J. Colwell, D. Lee, J. Soule, P. Lindsey, M. Bracey, A. Farr, S. Elsing, T. Thompson, J. Selby, T. Lyons, S. Yacoub‐Wasef, M. Szpiech, D. Wood, R. Mayfield, M. Molitch, D. Adelman, S. Colson, L. Jampol, A. Lyon, M. Gill, Z. Strugula, L. Kaminski, R. Mirza, E. Simjanoski, D. Ryan, C. Johnson, A. Wallia, S. Ajroud‐Driss, P. Astelford, N. Leloudes, A. Degillio, B. Schaefer, S. Mudaliar, G Lorenzi, M. Goldbaum, K. Jones, M. Prince, M. Swenson, I. Grant, R. Reed, R. Lyon, O. Kolterman, M. Giotta, T. Clark, G. Friedenberg, W.I. Sivitz, B. Vittetoe, J. Kramer, M. Bayless, R. Zeitler, H. Schrott, N. Olson, L. Snetselaar, R. Hoffman, J. MacIndoe, T. Weingeist, C. Fountain, R. Miller, S. Johnsonbaugh, M. Patronas, M. Carney, S. Mendley, P. Salemi, R. Liss, M. Hebdon, D. Counts, T. Donner, J. Gordon, R. Hemady, A. Kowarski, D. Ostrowski, S. Steidl, B. Jones, W.H. Herman, C.L. Martin, R. Pop‐Busui, D.A. Greene, M.J. Stevens, N. Burkhart, T. Sandford, J. Floyd, J. Bantle, N. Flaherty, J. Terry, D. Koozekanani, S. Montezuma, N. Wimmergren, B. Rogness, M. Mech, T. Strand, J. Olson, L. McKenzie, C. Kwong, F. Goetz, R. Warhol, D. Hainsworth, D. Goldstein, S. Hitt, J. Giangiacomo, D.S Schade, J.L. Canady, M.R. Burge, A. Das, R.B. Avery, L.H. Ketai, J.E. Chapin, M.L. Schluter, J. Rich, C. Johannes, D. Hornbeck, M. Schutta, P.A. Bourne, A. Brucker, S. Braunstein, S. Schwartz, B.J. Maschak‐Carey, L. Baker, T. Orchard, L. Cimino, T. Songer, B. Doft, S. Olson, D. Becker, D. Rubinstein, R.L. Bergren, J. Fruit, R. Hyre, C. Palmer, N. Silvers, L. Lobes, P. Paczan Rath, P.W. Conrad, S. Yalamanchi, J. Wesche, M. Bratkowksi, S. Arslanian, J. Rinkoff, J. Warnicki, D. Curtin, D. Steinberg, G. Vagstad, R. Harris, L. Steranchak, J. Arch, K. Kelly, P. Ostrosaka, M. Guiliani, M. Good, T. Williams, K. Olsen, A. Campbell, C. Shipe, R. Conwit, D. Finegold, M. Zaucha, A. Drash, A. Morrison, J.I. Malone, M.L. Bernal, P.R. Pavan, N. Grove, E.A. Tanaka, D. McMillan, J. Vaccaro‐Kish, L. Babbione, H. Solc, T.J. DeClue, S. Dagogo‐Jack, C. Wigley, H. Ricks, A. Kitabchi, E. Chaum, M.B. Murphy, S. Moser, D. Meyer, A. Iannacone, S. Yoser, M. Bryer‐Ash, S. Schussler, H. Lambeth, P. Raskin, S. Strowig, M. Basco, S. Cercone, B. Zinman, A. Barnie, R. Devenyi, M. Mandelcorn, M. Brent, S. Rogers, A. Gordon, N. Bakshi, B. Perkins, L. Tuason, F. Perdikaris, R. Ehrlich, D. Daneman, K. Perlman, S Ferguson, J. Palmer, R. Fahlstrom, I.H. de Boer, J. Kinyoun, L. Van Ottingham, S. Catton, J. Ginsberg, C. McDonald, J. Harth, M. Driscoll, T. Sheidow, J. Mahon, C. Canny, D. Nicolle, P. Colby, J. Dupre, I. Hramiak, N.W. Rodger, M. Jenner, T. Smith, W. Brown, M. May, J. Lipps Hagan, A. Agarwal, T. Adkins, R. Lorenz, S. Feman, L. Survant, N.H. White, L. Levandoski, G. Grand, M. Thomas, D. Joseph, K. Blinder, G. Shah, D. Burgess, I. Boniuk, J. Santiago, W. Tamborlane, P. Gatcomb, K. Stoessel, P. Ramos, K. Fong, P. Ossorio, J. Ahern, R. Gubitosi‐Klug, L. Meadema‐Mayer, C. Beck, K. Farrell, S. Genuth, J Quin, P. Gaston, M. Palmert, R. Trail, W. Dahms, J. Lachin, J. Backlund, I. Bebu, B. Braffett, L. Diminick, X. Gao, W. Hsu, K. Klumpp, H. Pan, V. Trapani, P. Cleary, P. McGee, W. Sun, S. Villavicencio, K. Anderson, L. Dews, Naji Younes, B. Rutledge, K. Chan, D. Rosenberg, B. Petty, A. Determan, D. Kenny, C. Williams, C. Cowie, C. Siebert, M. Steffes, V. Arends, J. Bucksa, M. Nowicki, B. Chavers, D. O'Leary, J. Polak, A. Harrington, L. Funk, R Crow, B. Gloeb, S. Thomas, C. O'Donnell, E.Z. Soliman, Z.M. Zhang, Y. Li, C. Campbell, L. Keasler, S. Hensley, J. Hu, M. Barr, T. Taylor, R. Prineas, E.L. Feldman, J.W. Albers, P. Low, C. Sommer, K. Nickander, T. Speigelberg, M. Pfiefer, M. Schumer, M. Moran, J. Farquhar, C. Ryan, D. Sandstrom, T. Williams, M. Geckle, E. Cupelli, F. Thoma, B. Burzuk, T. Woodfill, R. Danis, B. Blodi, D. Lawrence, H. Wabers, S. Gangaputra, S. Neill, M. Burger, J. Dingledine, V. Gama, R. Sussman, M. Davis, L. Hubbard, M. Budoff, S. Darabian, P. Rezaeian, N. Wong, M. Fox, R. Oudiz, L Kim, R. Detrano, K. Cruickshanks, D. Dalton, K. Bainbridge, J. Lima, D. Bluemke, E. Turkbey, . der Geest, C. Liu, A. Malayeri, A. Jain, C. Miao, H. Chahal, R. Jarboe, D.M. Nathan, V. Monnier, D. Sell, C. Strauch, S. Hazen, A. Pratt, W. Tang, J. Brunzell, J. Purnell, R. Natarajan, F. Miao, L. Zhang, Z. Chen, A. Paterson, A. Boright, S. Bull, L. Sun, S. Scherer, M. Lopes‐Virella, T.J. Lyons, A. Jenkins, R. Klein, G. Virella, A. Jaffa, R. Carter, J. Stoner, W.T. Garvey, D. Lackland, M. Brabham, D. McGee, D. Zheng, R.K. Mayfield, J. Maynard, H. Wessells, A Sarma, A. Jacobson, R. Dunn, S. Holt, J. Hotaling, C. Kim, Q. Clemens, J. Brown, K. McVary

**Affiliations:** ^1^ Department of Cellular and Molecular Medicine Lerner Research Institute Cleveland Clinic Cleveland OH; ^2^ Department of Cardiovascular Medicine Heart and Vascular Institute Cleveland Clinic Cleveland OH; ^3^ The Biostatistics Center George Washington University Rockville MD; ^4^ Cleveland Clinic (Emeritus) Cleveland OH

**Keywords:** diabetes mellitus, F2Isoprostane, free radical, paraoxonase, Biomarkers, Oxidant Stress, Inflammation

## Abstract

**Background:**

Hyperglycemia leading to increased oxidative stress is implicated in the increased risk for the development of macrovascular and microvascular complications in patients with type 1 diabetes mellitus.

**Methods and Results:**

A random subcohort of 349 participants was selected from the DCCT/EDIC (Diabetes Control and Complications Trial/Epidemiology of Diabetes Interventions and Complications) cohort. This included 320 controls and 29 cardiovascular disease cases that were augmented with 98 additional known cases to yield a case cohort of 447 participants (320 controls, 127 cases). Biosamples from DCCT baseline, year 1, and closeout of DCCT, and 1 to 2 years post‐DCCT (EDIC years 1 and 2) were measured for markers of oxidative stress, including plasma myeloperoxidase, paraoxonase activity, urinary F_2α_ isoprostanes, and its metabolite, 2,3 dinor‐8 *iso* prostaglandin F_2α_. Following adjustment for glycated hemoblobin and weighting the observations inversely proportional to the sampling selection probabilities, higher paraoxonase activity, reflective of antioxidant activity, and 2,3 dinor‐8 *iso* prostaglandin F_2α_, an oxidative marker, were significantly associated with lower risk of cardiovascular disease (−4.5% risk for 10% higher paraoxonase, *P*<0.003; −5.3% risk for 10% higher 2,3 dinor‐8 *iso* prostaglandin F_2α_, *P*=0.0092). In contrast, the oxidative markers myeloperoxidase and F_2α_ isoprostanes were not significantly associated with cardiovascular disease after adjustment for glycated hemoblobin. There were no significant differences between DCCT intensive and conventional treatment groups in the change in all biomarkers across time segments.

**Conclusions:**

Heightened antioxidant activity (rather than diminished oxidative stress markers) is associated with lower cardiovascular disease risk in type 1 diabetes mellitus, but these biomarkers did not change over time with intensification of glycemic control.

**Clinical Trial Registration:**

URL: https://www.clinicaltrials.gov. Unique identifiers: NCT00360815 and NCT00360893.

**Clinical Perspective**

**What Is New?**

- We observed that heightened antioxidant activity (as measured by paraoxonase‐1 activity), rather than diminished markers of oxidative stress, is associated with lower cardiovascular disease risk in patients with type 1 diabetes mellitus.
- We also observed that none of the biomarkers improved over time with intensification of glycemic control.

**What Are the Clinical Implications?**

- Hyperglycemia has been associated with increased oxidative stress, which has long been implicated in the increased risk for the development of macrovascular and microvascular complications in patients with type 1 diabetes mellitus.
- These findings challenged past reports and suggest that intrinsic counter‐regulatory mechanisms such as paraoxonase‐1 activity may play an important role in cardioprotection in patients with type 1 diabetes mellitus.

The DCCT (Diabetes Control and Complications Trial) and its observational follow‐up study, the EDIC (Epidemiology of Diabetes Interventions and Complications), showed that intensive therapy to lower glycated hemoglobin (HbA1c) to near‐normal concentrations reduces the risk of any cardiovascular disease (CVD) event in patients with type 1 diabetes mellitus (T1DM).^1^, ^2^, ^3^ Despite the landmark study's conclusion that intensive therapy has long‐term beneficial effects on the risk of CVD,^4^ the precise mechanisms by which cardiovascular complications of diabetes mellitus occur are still not well understood. Early studies have focused on the changes in lipoprotein levels as biomarkers for diabetes mellitus complications; specifically, oxidative modification of low‐density lipoprotein is believed to play a causative role in the development of atherosclerotic disease as well as microvascular complications of T1DM.^5^, ^6^, ^7^, ^8^, ^9^ Although lipoprotein markers have proved to be important for assessing cardiovascular risk, they do not completely account for the excess cardiovascular risk in diabetes mellitus.

Hyperglycemia leading to increased oxidative stress has been implicated as a key pathophysiological factor of macrovascular complications in T1DM.^10^ Through the use of specific measures of oxidative stress in the setting of known CVD‐reducing therapies such as 3‐hydroxy‐3‐methylglutaryl‐coenzyme A reductase inhibitors, strong correlations between distinct oxidative pathways and atherosclerotic disease have been established in the general population.^11^ Leukocyte‐derived oxidant production is triggered by granulocyte peroxidases such as myeloperoxidase (MPO).^12^ Furthermore, reactive species produced by activated neutrophils and other sources can lead to lipid peroxidation of the cellular membrane and the production of prostaglandin‐like compounds such as F_2_‐isoprostane (F_2_IP) and its major metabolite, 2,3 dinor‐8 iso prostaglandin F_2α_ (2,3‐dinor‐iPF_2α_‐III).^13^, ^14^, ^15^ Conversely, important high‐density lipoprotein components such as paraoxonases (PON) are antioxidants that can prevent the accumulation of lipid peroxides.^16^

Finally, the widely distributed antioxidant enzyme, PON1, is closely associated with HDL.^17^ Low levels of PON1 have consistently been associated with susceptibility to coronary heart disease in various case‐control studies.^18^, ^19^ Additionally, mouse models of PON1 overexpression have suggested the ability of PON to inhibit development of atherosclerosis in metabolic syndrome.^20^, ^21^ Because these associations have not been fully explored in T1DM, we assess the link between oxidative pathways and the risk for CVD as well as its relationship to chronic glycemic control in a longitudinal case‐cohort substudy of the DCCT/EDIC patient population.

## Methods

The data, analytical methods, and study materials will be/have been made available to other researchers through the National Institute of Diabetes and Digestive and Kidney Disease Data Repository for purposes of reproducing the results or replicating the procedure.^22^ These include the multiply imputed data sets that were constructed to account for missing data and the computer programs used to generate the tables herein.

### Study Population

The DCCT was a randomized controlled trial of 1441 patients who were between the ages of 13 and 39 years. Approximately half the subjects were enrolled in a primary prevention cohort with no pre‐existing microvascular complications and 1 to 5 years’ duration of T1DM, and half to a secondary intervention cohort with mild pre‐existing retinopathy, possible microalbuminuria, and 1 to 15 years’ duration preceding study entry.^23^ At baseline, patients with a history of CVD, hypertension (blood pressure of 140/90 mm Hg or more), or hypercholesterolemia (fasting serum cholesterol 3 SDs above age‐ and sex‐specific means) were excluded. Participants were assigned to either intensive therapy aimed at achieving chronic glycemic levels as close to the nondiabetic range as safely possible, or to conventional therapy that aimed to avoid symptomatic hyperglycemia and hypoglycemia. The cohort was followed for an average of 6.5 years. Following DCCT, 97% of the surviving original cohort agreed to join the observational follow‐up study, the EDIC. The DCCT and EDIC studies were approved by the institutional review boards of all participating institutions, and each participant provided written informed consent.

### Case‐Cohort Design

A case‐cohort design was used to assess the association of oxidative biomarkers of interest with CVD cases who had experienced an incident CVD event during the DCCT or during the first 16 years of follow‐up in EDIC.^1^ CVD events were defined as fatal or nonfatal myocardial infarction or stroke and death judged to be attributed to CVD, subclinical myocardial infarction present on an annual ECG, angina confirmed by ischemic changes on exercise tolerance testing, or by clinically significant obstruction on coronary angiography or coronary bypass.

A sample of 125 cases with a sample of 250 control subjects would provide 85% power to detect a hazard ratio (approximately equal to an odds ratio) of 1.39 per SD difference using a score test in the stratified Cox regression model at the 0.05 level 2‐sided.^24^

At the time that the sample was selected, a total of 127 subjects had experienced at least 1 CVD event (cases). A subcohort of 350 subjects was randomly selected from the DCCT cohort of 1441 subjects. No specimens were available for 1 patient, who was deleted. This subcohort included 29 CVD cases, leaving 320 controls without past CVD. To this, we added the remaining 98 then‐known CVD cases, yielding a study sample of 447 subjects (127 CVD cases+320 controls).

### Biospecimens

Patient blood and urine samples were collected as part of the DCCT/EDIC study. Oxidative stress markers were measured on stored biosamples from 4 time points: DCCT baseline, DCCT year 1, DCCT closeout (mean 6.5 years of treatment), and EDIC years 1 to 2.

### Laboratory Analysis

HbA1c was measured quarterly during the DCCT and annually during EDIC using a high‐precision, high‐performance liquid chromatography assay with long‐term control subjects to monitor assay stability as described.^25^ During DCCT, fasting lipid and serum creatinine levels and other risk factors for CVD were measured annually. During EDIC, fasting lipid levels and renal function were measured in alternate years.

Serum paraoxonase activity was determined using spectrophotometry in a 96‐well plate format (Spectramax 384 Plus; Molecular Devices, Sunnyvale, CA) as previously described.^24^ Rate of generation of paranitrophenol was determined at 405 nm in 40‐fold diluted serum (final) in reaction mixtures composed of 1.5 mmol/L of paraoxon (Sigma‐Aldrich, St. Louis, MO), 10 mmol/L of Tris hydrocholoride, pH 8, 1 mol/L of sodium chloride, and 2 mmol/L of calcium chloride at 24°C. An extinction coefficient (at 405 nm) of 17 000 mol/L/cm was used for calculating units of paraoxonase activity, which is expressed as nmol/L of paranitrophenol produced per minute per milliliter of serum. The intra‐assay and interassay coefficients of variance (CVs) for the high‐throughput PON1 activity assay were 1.9% and 3.3%, respectively, on 30 replicates performed on 15 different days. Plasma concentrations of MPO were determined by MPO assay on a Siemens Dimension XPand analyzer for EDTA plasma, with intra‐assay CV of 2.2% and interassay CV of 3.5%. Quantitation of urine metabolites F_2_‐IP and 2,3‐dinor‐iPF_2α_‐III were measured by stable isotope dilution liquid chromatography/tandem mass spectrometry analysis as previously described.^26^ The interday assay CV reported for the 2 assays averaged 10%, whereas the interday assay CV averaged 10.6%.^26^

### Statistical Analysis

Owing to depletion of saved specimens from past DCCT/EDIC ancillary studies, 261 of the 1759 (15%) expected samples were missing, 255 owing to depletion and only 6 owing to loss to follow‐up. To address these missing data, multiple imputation was used to provide 10 estimates of each missing value and yielding 10 complete data sets.^27^ A given analysis was then repeated using each of the 10 complete data sets, and the results were averaged using the methods of Rubin and Schenker.^28^ The resulting confidence limits and *P* values accounted for the overall extent of the missing original data.

A survey sampling analysis using weights inversely proportional to the sampling probability was used to describe the characteristics of the weighted subsample in comparison to the full cohort.^29^ Mean values of the biomarkers over time with tests of treatment group differences were obtained using survey sampling regression models.^29^

Separate analyses of the association of each natural log‐transformed biomarker with the risk of an initial CVD event were conducted using Barlow's modified Cox proportional hazards model for application to a case‐cohort design that used the survey weights equal to the inverse of the subcohort sampling probabilities.^30^, ^31^ Cox proportional hazards models within each group were stratified by baseline cohort and the models in the combined groups were also stratified by treatment group. All associations are presented as the percent change in CVD risk per 10% increase in the biomarker, with and without adjustment for HbA1c as a time‐dependent covariate. The biomarker entered the model as a time‐dependent covariate with the biomarker value at each of the 4 measurement times. The time‐dependent HbA1c covariate value used the eligibility screening value at baseline, the mean over the first year at year 1, the mean up to closeout at closeout, and the mean over DCCT and EDIC up to EDIC year 1 or 2. Additional models tested group by biomarker interaction effects.

The Appendix S1 provides a technical description of the statistical methods and the software that were used.

## Results

The weighted estimates of patient characteristics in the total case‐cohort sample (N=447) were similar to the aggregate estimates in the original complete DCCT cohort (N=1441), and the validity of the inverse probability weighted analyses was verified (Table 1).

**Table 1:** Baseline Characteristics of the DCCT/EDIC Case‐Control and Total Cohorts

	Weighted Case‐Control Cohort	Total DCCT Cohort
N	447	1441
Age, y	26.7±7.3	26.8±7.1
Female, %	46	47
Primary prevention cohort, %*	51	50
Intensive treatment group, %	49	49
Diabetes mellitus duration, mo	69.2±50.9	67.6±49.9
Body mass index, kg/m^2^	23.5±2.7	23.5±2.8
HbA1c, %	8.8±1.5	8.9±1.6
HbA1c, mmol/mol	73.2±16.4	74.0±17.5

Unless otherwise indicated, data are means±SD or % estimated using the survey sample weights based on the inverse sampling probabilities within strata defined by primary vs secondary cohort, intensive vs conventional treatment, and CVD case vs control. CVD indicates cardiovascular disease; DCCT, Diabetes Control and Complications Trial; EDIC, Epidemiology of Diabetes Interventions and Complications; HbA1c, glycated hemoglobin.

Primary prevention cohort and secondary intervention cohort are based on the original DCCT study design, see Methods.

Table 2 describes the mean values of the biomarkers over the selected study period, with tests of significant differences within and across treatment groups and points in time. A significant difference (*P*<0.05) between baseline and DCCT year 1 was observed within both the intensive and conventional treatment groups (separately) for 2,3‐dinor‐iPF_2α_‐III and for F_2_IP, but with no significant difference between groups. Changes between DCCT closeout and EDIC year 1 to 2 were not significant within either treatment group and did not differ between groups.

**Table 2:** Mean Values of Biomarkers Over the Study Period (DCCT Baseline, DCCT year 1, Closeout DCCT, and EDIC Year 1–2)

	DCCT Baseline	DCCT Year 1	Difference	Closeout DCCT	EDIC Year 1 to 2	Difference
MPO (pmol/L)						
Intensive	405.1±21.6	359.4±19.7	−45.7 (−97.2, 5.9) *P*=0.08	383.15±18.5	351.0±15.8	−32.13 (−74.5, 10.2) *P*=0.1364
Conventional	359.97±18.0	374.8±20.7	14.8 (−31.8, 61.4) *P*=0.53	368.4±15.8	390.4±21.8	22.0 (−20.0, 64.1) *P*=0.3044
Intensive vs conventional			−60.5 (−131.1, 10.2) *P*=0.0929			−54.2 (−112.2, 3.9) *P*=0.0675
PON activity, μmol/min/mL						
Intensive	912.2±47.1	895.1±44.6	−17.1 (−55.7, 21.6) *P*=0.3838	884.3±47.7	884.9±49.0	0.64 (−57.8, 59.1) *P*=0.9823
Conventional	771.3±40.9	765.4±39.4	−5.9 (−38.7, 26.9) *P*=0.7226	747.1±40.4	754.2±51.1	7.03 (−66.4, 80.5) *P*=0.8466
Intensive vs conventional			−11.14 (−63.0, 40.7) *P*=0.6716			−6.4 (−100.01, 87.2) *P*=0.8906
F2α‐isoprostane (pg/mg Cr)						
Intensive	1.16±0.05	1.31±0.06	0.15 (0.04, 0.26) *P*=0.0074	1.49±0.084	1.46±0.062	−0.02 (−0.19, 0.14) *P*=0.7699
Conventional	1.12±0.054	1.31±0.07	0.19 (0.07, 0.31) *P*=0.0023	1.27±0.063	1.30±0.063	0.04 (−0.09, 0.17) *P*=0.5534
Intensive vs conventional			−0.04 (−0.19, 0.12) *P*=0.6191			−0.06 (−0.28, 0.16) *P*=0.5699
2,3‐dinor‐iPF2α‐III (pg/mg Cr)						
Intensive	9.86±0.51	12.7±0.94	2.84 (0.85, 4.83) *P*=0.0064	13.7±0.99	13.22±0.90	−0.46 (−2.57, 1.65) *P*=0.6649
Conventional	9.93±0.46	11.44±0.76	1.51 (0.1, 2.93) *P*=0.037	11.51±0.61	11.9±0.713	0.37 (−0.87, 1.61) *P*=0.5551
Intensive vs conventional			1.33 (−1.4, 4.05) *P*=0.3267			−0.83 (−3.1, 1.44) *P*=0.4707

Unadjusted means are presented in the table with standard errors. Differences within each treatment group for the time periods (baseline to DCCT year 1, and DCCT closeout to EDIC year 1–2) with 95% confidence intervals are shown. 2,3‐dinor‐iPF_2α_‐III indicates 2,3 dinor‐8 *iso* prostaglandin F_2α_; DCCT, Diabetes Control and Complications Trial; EDIC, Epidemiology of Diabetes Interventions and Complications; MPO, myeloperoxidase; PON, paraoxonase.

Table 3 presents the association of each biomarker (natural log‐transformed) as a time‐dependent covariate with the risk of CVD separately within the intensive and conventional groups, and the 2 combined, without and with adjustment for time‐dependent HbA1c. After adjustment for time‐dependent HbA1c, for a 10% increase in PON, the risk of CVD lowers by 4.5% (*P*=0.0026) in the total cohort, and by 4.6% (*P*=0.0125) in the conventional group. Similar effects were observed without the HbA1c adjustment, as well as a nominally significant reduction by 4.8% in the intensive group (*P*=0.046).

**Table 3:** Association of Biomarker With Risk of CVD Separately Within Each Treatment Group and Combined, With and Without Adjustment for HbA1c As a Time‐Dependent Covariate^a^

Biomarker	Intervention	Nonadjusted for HbA1c			Adjusted for HbA1C		
		% Change in Risk for a 10% Higher Value (95% CI)	*P* Value	Model Chi Square	% Change in Risk for a 10% Higher Value (95% CI)	*P* Value	Model Chi Square
MPO	Intensive	4.2 (−2.3, 11.1)	0.21	2.2	4.0 (−2.3, 10.8)	0.22	6.5
	Conventional	2.2 (−2.2, 6.9)	0.33	1.3	2.9 (−1.8, 7.8)	0.23	9.72
	Total	2.9 (−0.8, 6.8)	0.12	3.08	3.3 (−0.4, 7.2)	0.083	15.97
PON activity	Intensive	−4.8 (−9.3, −0.1)	0.046	5.9	−4.3 (−8.9, 0.6)	0.09	8.8
	Conventional	−4.9 (−8, −1.6)	0.0036	10.3	−4.6 (−8.1, −1)	0.0125	16.9
	Total	−4.9 (−7.4, −2.2)	0.0004	16.1	−4.5 (−7.3, −1.6)	0.0026	25.5
F_2_IP	Intensive	−10.2 (−19.8, 0.6)	0.06	4.9	−9.4 (−19.4, 1.8)	0.097	8.54
	Conventional	−6.6 (−15.6, 3.3)	0.18	2.9	−6.1 (−15.2, 4.0)	0.23	10.2
	Total	−8.1 (−15, −0.7)	0.034	7.4	−7.5 (−14.6, 0.2)	0.057	18.4
2,3‐dinor‐iPF_2α_‐III	Intensive	−6.1 (−11.0, −0.8)	0.02	5.4	−6.4 (−11.7, −0.8)	0.026	10.5
	Conventional	−4.6 (−10.0, 1.1)	0.11	4.1	−4.5 (−9.9, 1.2)	0.12	11.6
	Total	−5.2 (−9, −1.4)	0.0086	9.1	−5.3 (−9.1, −1.4)	0.0092	21.6

2,3‐dinor‐iPF_2α_‐III indicates 2,3 dinor‐8 *iso* prostaglandin F_2α_; CI, confidence interval; CVD, cardiovascular disease; F_2_IP, F_2_‐isoprostane; HbA1c, glycated hemoglobin; MPO, myeloperoxidase; PON, paraoxonase.

a All analyses used the log (biomarker). To assess the percent change in risk for a higher 10% value in the biomarker, the formula used is: 100×(1.1^β−1^).

For a 10% increase in 2,3‐dinor‐iPF2α‐III, the risk of CVD lowers by 5.3% (*P*=0.0092) in the total cohort, and by 6.4% (*P*=0.026) in the intensive group, with similar associations without the HbA1c adjustment. There was a marginally significant 8.1% reduction in CVD risk with a 10% increase in F_2_IP (*P*=0.034) without adjustment for HbA1c that became nonsignificant after such adjustment (*P*=0.057). In contrast, MPO was not significantly associated with CVD in either group or total, without or with adjustment for HbA1c.

In general, the effect sizes within each group are similar, if not greater, than in total; however, the *P* values within each group could be nonsignificant owing to the smaller sample size within each group. A test of interaction did not show any significant differences in the biomarker effects between groups.

## Discussion

Strong evidence has tied oxidative stress to the development of CVD in the general population. In this study, we utilize biomarkers that are distinct elements of the oxidative process. There are several novel findings in this study. First, we observed that none of our oxidative stress biomarkers changed over time in either treatment group and did not differ significantly between groups during the course of the study. Second, increases in all but MPO were associated with lower risk of CVD with and without adjustment for HbA1c as a time dependent covariate (marginally for F_2_IP after adjustment). These results suggest that despite their associations with lower CVD risk, biomarkers of oxidative stress are largely unaffected by intensification of glycemic control in this prospectively randomized study of patients with T1DM. These findings are surprising given previous DCCT/EDIC studies showing increased oxidized low‐density lipoprotein and advanced glycation end products as predictors of increased coronary artery calcification or carotid artery intimamedial thickness.^5^, ^7^, ^8^ The discrepancy may suggest different mechanisms by which modified low‐density lipoprotein forms contribute to disease beyond those reflected by our panel of markers. Furthermore, carotid artery intimamedial thickness and coronary artery calcification are surrogate measures of atherosclerosis and do not directly indicate clinical events. For example, carotid intimamedial thickness is not recommended for routine measurement in clinical practice for risk assessment for a first atherosclerotic CVD event by recent American College of Cardiology/American Heart Association guidelines,^32^ whereas coronary artery calcification scores are generally weighted upward for greater calcium density. However, more‐recent data have suggested that increased plaque calcium density may be inversely correlated with CVD risk.^33^

Similar to the oxidative marker findings, we observed an association between higher antioxidant PON activity and lower CVD risk in this cohort, especially in the conventional treatment group, both unadjusted and adjusted for HbA1c. An additional analysis (not shown) adjusted for high‐density lipoprotein levels and smoking status at the time of each biomarker measurement provided concordant results.

Diminished PON activity has been linked with T1DM in several studies.^34^, ^35^, ^36^, ^37^, ^38^ Paraoxonases constitute a family of calcium‐dependent esterases with 3 isoforms: PON1, PON2, and PON3, with PON1 being the primary form found in serum.^39^ Whereas all 3 isoforms exhibit arylesterase and paraoxonase activities, the native enzymatic activity of PON is thought to be as a lactonase that may modify several prodrugs.^39^ Studies have shown that both the more‐abundant PON1, as well as PON3, bind to the high‐density lipoprotein particle and circulate.^40^ These pleiotropic enzymes are genetically highly conserved across species, and their diverse roles include protection against lipid peroxidation and oxidative stress, modulation against endoplasmic reticulum stress, and regulation of cell proliferation and apoptosis.^41^ Diminished serum PON paraoxonase and arylesterase activities have been directly associated with increased circulating levels of structurally defined specific oxidized fatty acids^42^ and may provide incremental prognostic value in stable cardiac patients, even among those with no significant coronary artery stenosis by angiography who might otherwise be dismissed as low risk.^24^ Meanwhile, serum PON activity levels strongly tracked with genetic polymorphisms linked to PON1 genotype (especially Q192R), thus confirming the contribution of PON1 in these esterase activities. It has been reported that glycation and glycoxidation of PON1 substantially reduces the ability for PON1 to metabolize membrane lipid hydroperoxides in vitro.^43^ Therefore, it is interesting that PON1 did not appear to attenuate long‐term CVD risk despite the intensification of glycemic control in T1DM given the similar mechanisms of hemoglobin and PON glycation.

Enhanced oxidative stress as a result of free radical lipid peroxidation can be measured by increased levels of F_2_IP, which has long been considered 1 of the gold standards for the assessment of in vivo oxidative stress.^15^, ^44^ Also, F_2_IP and its metabolites, such as 2,3‐dinor‐iPF_2α_‐III, have been consistently found to be higher in smokers.^45^ However, despite that the majority of studies that have been performed on F_2_IP as a risk marker have been cross‐sectional,^46^ a relationship between F_2_IP and atherosclerotic risk has been established.^47^ In contrast, lower 2, 3‐dinor‐iPF_2α_‐III (and to some extent F_2_IP) was associated with higher CVD risk in DCCT/EDIC. These unexpected observations were inconsistent with the association between these indices of oxidative stress and glycemic control in children and adolescents with T1DM.^48^, ^49^, ^50^ Furthermore, our findings did not support the notion that intensification of glycemic control confers any improvement in F_2_IP and 2,3‐dinor‐iPF_2α_‐III levels over time. Whereas in T1DM, insulin action may contribute to enhancement in antioxidant effects and help explain the stability of the observed oxidative markers over time, we do not find it likely that this actually plays a large role in our study. In a previous assessment of risk factors for CVD and major atherosclerotic cardiovascular events, daily insulin dose did not emerge in the final model for CVD or major atherosclerotic cardiovascular events. Insulin dose alone did have a nominally significant association with risk of any CVD, but was not significant in a model with other factors.^51^

In contrast to the F_2_IP formed as a product of numerous oxidative mechanisms, MPO serves as a catalytic source of specific oxidative processes often associated with inflammation, including generation of reactive nitrogen and chlorinating species. Multiple studies have found MPO to be a prognostic marker for CVD risk.^12^, ^52^, ^53^, ^54^, ^55^, ^56^ Furthermore, MPO has been reported to be part of the causal pathway in atherogenesis by its ability to initiate lipid oxidation and render low‐density lipoprotein modified into a form that fosters macrophage scavenger receptor recognition, cholesterol accumulation, and foam cell formation.^57^ Interestingly, there has been a paucity of studies on MPO in T1DM. In a small study of 30 children with T1DM, MPO levels were significantly higher than age‐matched controls, as well as associated with atherosclerosis‐related structural and functional changes of the arterial wall.^58^ The lack of association between MPO and incident CVD risk in the DCCT/EDIC cohort, even in the unadjusted model, is therefore unexpected.

Our findings in this study differ compared with previous studies and challenge the common belief of elevated oxidative markers as harmful stressors. However, recent evidence is evolving the interpretation of these markers. Prospective analysis of urinary F_2_IP and its metabolites has also found an inverse association with the risk of developing type 2 diabetes mellitus, a group known to be highly susceptible to CVD.^59^ As previously mentioned, a majority of evidence linking F_2_IP to CVD has been cross‐sectional. Thus, the contrasting finding between prospective and cross‐sectional elevation of F_2_IP suggests dynamic effects over time that may not be captured through cross‐sectional studies. These observations suggest a need for re‐evaluation of how we interpret systemic oxidative markers. Citing a recent hypothesis that CVD and diabetes mellitus, in part, may be mediated by the failure to generate sufficient reactive oxygen species,^60^ the inverse relationship between F_2_IP and CVD risk in our study can be explained by measurement of F_2_IP over time as a marker of systemic metabolic compensation. These oxidative marker findings appear to contrast with the observation that increased antioxidant PON activity is associated with lower CVD risk, but they may, in fact, represent the balancing act of disease modulation. However, only additional mechanistic studies will help expand our understanding. Overall, our findings present a novel view of biomarker associations in the T1DM population with a dominant pattern of higher oxidative stress marker along with increased antioxidant activity associated with diminished risk of CVD.

## Conclusions

In the DCCT/EDIC study, heightened antioxidant activity (as measured by PON activity), rather than diminished markers of oxidative stress, is associated with lower CVD risk in T1DM. None of the biomarkers improved over time with intensification of glycemic control.

## Appendix

### The DCCT/EDIC Research Group

The members of the DCCT/EDIC Research Group at the time of this publication follow:

Study Chairpersons—D.M. Nathan, B. Zinman (vice‐chair), O. Crofford (past), S. Genuth (past).

#### Clinical Centers

Albert Einstein College of Medicine—J. Brown‐Friday (past), J. Crandall (past), H. Engel (past), S. Engel (past), H. Martinez (past), M. Phillips (past), M. Reid (past), H. Shamoon (past), J. Sheindlin (past).

Case Western Reserve University—R. Gubitosi‐Klug, L. Mayer, S. Pendegast, H. Zegarra, D. Miller, L. Singerman, S. Smith‐Brewer, M. Novak, J. Quin (past), Saul Genuth (past), M. Palmert (past), E. Brown (past), J. McConnell (past), P. Pugsley (past), P. Crawford (past), W. Dahms (deceased).

Weill Cornell Medical College—N.S. Gregory, M.E. Lackaye, S. Kiss, R. Chan, A. Orlin, M. Rubin, D. Brillon (past), V. Reppucci (past), T. Lee (past), M. Heinemann (past), S. Chang (past), B. Levy (past), L. Jovanovic (past), M. Richardson (past), B. Bosco (past), A. Dwoskin (past), R. Hanna (past), S. Barron (past), R. Campbell (deceased).

Henry Ford Health System—A. Bhan, D. Kruger, J.K. Jones, P.A. Edwards, A. Bhan, J.D. Carey, E. Angus, A. Thomas, A. Galprin (past), M. McLellan (past), F. Whitehouse (past).

International Diabetes Center—R. Bergenstal, M. Johnson, K. Gunyou, L. Thomas, J. Laechelt, P. Hollander (past), M. Spencer (past), D. Kendall (past), R. Cuddihy (past), P. Callahan (past), S. List (past), J. Gott (past), N. Rude (past), B. Olson (past), M. Franz (past), G. Castle (past), R. Birk (past), J. Nelson (past), D. Freking (past), L. Gill (past), W. Mestrezat (past), D. Etzwiler (deceased), K. Morgan (deceased).

Joslin Diabetes Center—L.P. Aiello, E. Golden, P. Arrigg, V. Asuquo, R. Beaser, L. Bestourous, J. Cavallerano, R. Cavicchi, O. Ganda, O. Hamdy, R. Kirby, T. Murtha, D. Schlossman, S. Shah, G. Sharuk, P. Silva, P. Silver, M. Stockman, J. Sun, E. Weimann, H. Wolpert, L.M. Aiello (past), A. Jacobson (past), L. Rand (past), J. Rosenzwieg (past).

Massachusetts General Hospital—D.M. Nathan, M.E. Larkin, M. Christofi, K. Folino, J. Godine, P. Lou, C. Stevens, E. Anderson (past), H. Bode (past), S. Brink (past), C. Cornish (past), D. Cros (past), L. Delahanty (past), A. deManbey (past), C. Haggan (past), J. Lynch (past), C. McKitrick (past), D. Norman (past), D. Moore (past), M. Ong (past), C. Taylor (past), D. Zimbler (past), S. Crowell (past), S. Fritz (past), K. Hansen (past), C. Gauthier‐Kelly (past).

Mayo Clinic—F.J. Service, G. Ziegler, A. Barkmeier, L. Schmidt (past), B. French (past), R. Woodwick (past), R. Rizza (past), W.F. Schwenk (past), M. Haymond (past), J. Pach (past), J. Mortenson (past), B. Zimmerman (deceased), A. Lucas (deceased), R. Colligan (deceased).

Medical University of South Carolina—L. Luttrell, M. Lopes‐Virella, S. Caulder, C. Pittman, N. Patel, K. Lee, M. Nutaitis, J. Fernandes, K. Hermayer, S. Kwon, A. Blevins, J. Parker, J. Colwell (past), D. Lee (past), J. Soule (past), P. Lindsey (past), M. Bracey (past), A. Farr (past), S. Elsing (past), T. Thompson (past), J. Selby (past), T. Lyons (past), S. Yacoub‐Wasef (past), M. Szpiech (past), D. Wood (past), R. Mayfield (past).

Northwestern University—M. Molitch, D. Adelman, S. Colson, L. Jampol, A. Lyon, M. Gill, Z. Strugula, L. Kaminski, R. Mirza, E. Simjanoski, D. Ryan, C. Johnson, A. Wallia, S. Ajroud‐Driss, P. Astelford, N. Leloudes, A. Degillio, B. Schaefer (past).

University of California, San Diego—S. Mudaliar, G. Lorenzi, M. Goldbaum, K. Jones (past), M. Prince (past), M. Swenson (past), I. Grant (past), R. Reed (past), R. Lyon (past), O. Kolterman (past), M. Giotta (past), T. Clark (past), G. Friedenberg (deceased).

University of Iowa—W.I. Sivitz, B. Vittetoe, J. Kramer, M. Bayless (past), R. Zeitler (past), H. Schrott (past), N. Olson (past), L. Snetselaar (past), R. Hoffman (past), J. MacIndoe (past), T. Weingeist (past), C. Fountain (past).

University of Maryland School of Medicine—R. Miller, S. Johnsonbaugh, M. Patronas, M. Carney, S. Mendley (past), P. Salemi (past), R. Liss (past), M. Hebdon (past), D. Counts (past), T. Donner (past), J. Gordon (past), R. Hemady (past), A. Kowarski (past), D. Ostrowski (past), S. Steidl (past), B. Jones (past).

University of Michigan—W.H. Herman, C.L. Martin, R. Pop‐Busui, D.A. Greene (past), M.J. Stevens (past), N. Burkhart (past), T. Sandford (past), J. Floyd (deceased).

University of Minnesota—J. Bantle, N. Flaherty, J. Terry, D. Koozekanani, S. Montezuma, N. Wimmergren (past), B. Rogness (past), M. Mech (past), T. Strand (past), J. Olson (past), L. McKenzie (past), C. Kwong (past), F. Goetz (past), R. Warhol (past).

University of Missouri—D. Hainsworth, D. Goldstein, S. Hitt, J. Giangiacomo (deceased).

University of New Mexico—D.S. Schade, J.L. Canady, M.R. Burge, A. Das, R.B. Avery, L.H. Ketai, J.E. Chapin, M.L. Schluter (past) J. Rich (past), C. Johannes (past), D. Hornbeck (past).

University of Pennsylvania—M. Schutta, P.A. Bourne, A. Brucker, S. Braunstein (past), S. Schwartz (past), B.J. Maschak‐Carey (past), L. Baker (deceased).

University of Pittsburgh—T. Orchard, L. Cimino, T. Songer, B. Doft, S. Olson, D. Becker, D. Rubinstein, R.L. Bergren, J. Fruit, R. Hyre, C. Palmer, N. Silvers (past), L. Lobes (past), P. Paczan Rath (past), P.W. Conrad (past), S. Yalamanchi (past), J. Wesche (past), M. Bratkowksi (past), S. Arslanian (past), J. Rinkoff (past), J. Warnicki (past), D. Curtin (past), D. Steinberg (past), G. Vagstad (past), R. Harris (past), L. Steranchak (past), J. Arch (past), K. Kelly (past), P. Ostrosaka (past), M. Guiliani (past), M. Good (past), T. Williams (past), K. Olsen (past), A. Campbell (past), C. Shipe (past), R. Conwit (past), D. Finegold (past), M. Zaucha (past), A. Drash (deceased).

University of South Florida—A. Morrison, J.I. Malone, M.L. Bernal, P.R. Pavan, N. Grove, E.A. Tanaka (past), D. McMillan (past), J. Vaccaro‐Kish (past), L. Babbione (past), H. Solc (past), T.J. DeClue (past).

University of Tennessee—S. Dagogo‐Jack, C. Wigley, H. Ricks, A. Kitabchi, E. Chaum, M.B. Murphy (past), S. Moser (past), D. Meyer (past), A. Iannacone (past), S. Yoser (past), M. Bryer‐Ash (past), S. Schussler (past), H. Lambeth (past).

University of Texas Southwestern Medical Center at Dallas—P. Raskin, S. Strowig, M. Basco (past), S. Cercone (deceased).

University of Toronto—B. Zinman, A. Barnie, R. Devenyi, M. Mandelcorn, M. Brent, S. Rogers, A. Gordon, N. Bakshi, B. Perkins, L. Tuason, F. Perdikaris, R. Ehrlich (past), D. Daneman (past), K. Perlman (past), S. Ferguson (past).

University of Washington—J. Palmer, R. Fahlstrom, I.H. de Boer, J. Kinyoun, L. Van Ottingham, S. Catton (past), J. Ginsberg (past).

University of Western Ontario—C. McDonald, J. Harth, M. Driscoll, T. Sheidow, J. Mahon (past), C. Canny (past), D. Nicolle (past), P. Colby (past), J. Dupre (past), I. Hramiak (past), N.W. Rodger (past), M. Jenner (past), T. Smith (past), W. Brown (past).

Vanderbilt University—M. May, J. Lipps Hagan, A. Agarwal, T. Adkins, R. Lorenz (past), S. Feman (past), L. Survant (deceased).

Washington University, St. Louis—N.H. White, L. Levandoski, G. Grand, M. Thomas, D. Joseph, K. Blinder, G. Shah, D. Burgess (past), I. Boniuk (deceased), J. Santiago (deceased).

Yale University School of Medicine—W. Tamborlane, P. Gatcomb, K. Stoessel, P. Ramos, K. Fong, P. Ossorio, J. Ahern (past).

#### Clinical Coordinating Center

Case Western Reserve University—R. Gubitosi‐Klug, L. Meadema‐Mayer, C. Beck, K. Farrell, S. Genuth (past), J. Quin (past), P. Gaston (past), M. Palmert (past), R. Trail (past), W. Dahms (deceased).

#### Data Coordinating Center

George Washington University, The Biostatistics Center—J. Lachin, J. Backlund, I. Bebu, B. Braffett, L. Diminick, X. Gao, W. Hsu, K. Klumpp, H. Pan, V. Trapani, P. Cleary (past), P. McGee (past), W. Sun (past), S. Villavicencio (past), K. Anderson (past), L. Dews (past), Naji Younes (past), B. Rutledge (past), K. Chan (past), D. Rosenberg (past), B. Petty (past), A. Determan (past), D. Kenny (past), C. Williams (deceased).

#### National Institute of Diabetes and Digestive and Kidney Disease

National Institute of Diabetes and Digestive and Kidney Disease Program Office—C. Cowie, C. Siebert (past).

#### Central Units

Central Biochemistry Laboratory (University of Minnesota)—M. Steffes, V. Arends, J. Bucksa (past), M. Nowicki (past), B. Chavers (past).

Central Carotid Ultrasound Unit (New England Medical Center)—D. O'Leary, J. Polak, A. Harrington, L. Funk (past).

Central ECG Reading Unit (University of Minnesota)—R. Crow (past), B. Gloeb (past), S. Thomas (past), C. O'Donnell (past).

Central ECG Reading Unit (Wake Forest School of Medicine)—E.Z. Soliman, Z.M. Zhang, Y. Li, C. Campbell, L. Keasler, S. Hensley, J. Hu, M. Barr, T. Taylor, R. Prineas (past).

Central Neurologic Reading Center (University of Michigan, Mayo Clinic, Southern Illinois University)—E.L. Feldman (past), J.W. Albers (past), P. Low (past), C. Sommer (past), K. Nickander (past), T. Speigelberg (past), M. Pfiefer (past), M. Schumer (past), M. Moran (past), J. Farquhar (past).

Central Neuropsychological Coding Unit (University of Pittsburgh)—C. Ryan (past), D. Sandstrom (past), T. Williams (past), M. Geckle (past), E. Cupelli (past), F. Thoma (past), B. Burzuk (past), T. Woodfill (past).

Central Ophthalmologic Reading Center (University of Wisconsin)—R. Danis, B. Blodi, D. Lawrence, H. Wabers, S. Gangaputra (past), S. Neill (past), M. Burger (past), J. Dingledine (past), V. Gama (past), R. Sussman (past), M. Davis (past), L. Hubbard (past).

Computed Tomography Reading Center (Harbor UCLA Research and Education Institute)—M. Budoff, S. Darabian, P. Rezaeian, N. Wong (past), M. Fox (past), R. Oudiz (past), L. Kim (past), R. Detrano (past).

Audiometry Reading Center (EpiSense, University of Wisconsin)—K. Cruickshanks, D. Dalton, K. Bainbridge (National Institute on Deafness and Other Communication Disorders).

Cardiac MR Reading Center (Johns Hopkins University, National Heart Lung and Blood Institute)—J. Lima, D. Bluemke, E. Turkbey, R.J. van der Geest (past), C. Liu (past), A. Malayeri (past), A. Jain (past), C. Miao (past), H. Chahal (past), R. Jarboe (past).

Editor, EDIC Publications—D.M. Nathan.

#### Collaborators

Advanced Glycation End Products (Case Western Reserve University)—V. Monnier, D. Sell, C. Strauch.

Biomarkers (Cleveland Clinic)—S. Hazen, A. Pratt, W. Tang.

Central Obesity Study (University of Washington)—J. Brunzell, J. Purnell.

Epigenetics (Beckman Research Institute of City of Hope Medical Center)—R. Natarajan, F. Miao, L. Zhang, Z. Chen.

Genetic Studies (Hospital for Sick Children)—A. Paterson, A. Boright, S. Bull, L. Sun, S. Scherer (past).

Molecular Risk Factors Program Project (Medical University of South Carolina)—M. Lopes‐Virella, T.J. Lyons, A. Jenkins, R. Klein, G. Virella, A. Jaffa, R. Carter, J. Stoner, W.T. Garvey (past), D. Lackland (past), M. Brabham (past), D. McGee (past), D. Zheng (past), R.K. Mayfield (past).

SCOUT (Veralight)—J. Maynard (past).

UroEDIC (University of Washington, University of Michigan)—H. Wessells, A. Sarma, A. Jacobson, R. Dunn, S. Holt, J. Hotaling, C. Kim, Q. Clemens, J. Brown (past), K. McVary (past).

## Author Contributions

Dr Lachin and Ms McGee had full access to all of the data in the study and both take responsibility for the integrity of the data and the accuracy of the data analysis. Drs Tang and Hazen and Mr Li drafted the article. Drs Hoogwerf, Lachin, and Ms McGee helped revise the article. D.M. Nathan is the editor for DCCT/EDIC publications. Industry Support: Industry contributors have had no role in the DCCT/EDIC study, but have provided free or discounted supplies or equipment to support participants’ adherence to the study: Abbott Diabetes Care (Alameda, CA), Animas (Westchester, PA), Bayer Diabetes Care (North America Headquarters, Tarrytown, NY), Becton Dickinson (Franklin Lakes, NJ), Eli Lilly (Indianapolis, IN), Extend Nutrition (St. Louis, MO), Insulet Corporation (Bedford, MA), Lifescan (Milpitas, CA), Medtronic Diabetes (Minneapolis, MN), Nipro Home Diagnostics (Ft. Lauderdale, FL), Nova Diabetes Care (Billerica, MA), Omron (Shelton, CT), Perrigo Diabetes Care (Allegan, MI), Roche Diabetes Care (Indianapolis, IN), and Sanofi‐Aventis (Bridgewater NJ).

## Sources of Funding

The DCCT/EDIC has been supported by cooperative agreement grants (1982–1993, 2012–2017), and contracts (1982–2012) with the Division of Diabetes Endocrinology and Metabolic Diseases of the National Institute of Diabetes and Digestive and Kidney Disease (current grant numbers U01 DK094176 and U01 DK094157), and through support by the National Eye Institute, the National Institute of Neurologic Disorders and Stroke, the General Clinical Research Centers Program (1993–2007), and Clinical Translational Science Center Program (2006–present), Bethesda, MD. The research in this ancillary study to the DCCT/EDIC was supported by National Institutes of Health grants P01HL076491, R01HL128300, and 3R01DK080732‐01A1S1.

## Disclosures

Dr Hazen is named as inventor on pending and issued patents held by the Cleveland Clinic relating to cardiovascular diagnostics and therapeutics. Dr Hazen is a paid consultant for P&G. Dr Hazen has received research funds from P&G, Pfizer Inc., and Roche Diagnostics. Dr Hazen has received royalty payments for inventions or discoveries related to cardiovascular diagnostics or therapeutics from Cleveland HeartLab, P&G, Siemens, Esperion, and Frantz Biomarkers, LLC. The remaining authors have no disclosures to report.

## Supporting information

EDIC Subset Case‐cohort Analyses
