# Supplementary material for: Oxidative Stress and Cardiovascular Risk in Type 1 Diabetes Mellitus: Insights From the DCCT/EDIC Study
Source: J Am Heart Assoc. 2018 May 9;7(10):e008368. doi: 10.1161/JAHA.117.008368 (PMC6015340; doi:10.1161/JAHA.117.008368)
Supplement: Supplementary file 1 — EDIC Subset Case‐cohort Analyses [file JAH3-7-e008368-s001.pdf]

SUPPLEMENTAL MATERIAL

Supplemental Material:
EDIC Subset Case-cohort Analyses

Oxidative Stress and Cardiovascular Risk in Type 1 Diabetes Mellitus: Insights from the DCCT/EDIC Study

Writing Group: W. H. Wilson Tang, M.D., Paula McGee, M.S.,
John M. Lachin, Sc.D, Daniel Y. Li, B.S., Byron Hoogwerf M.D.,
Stanley L. Hazen, M.D. Ph.D., and the DCCT/EDIC Research Group

March 6, 2018

1 Case-Cohort Sampling

This study was designed to conduct 3 separate sub-studies of the association of biomarkers with the incidence of progression of retinopathy, progression of nephropathy, and of cardiovascular disease. For each outcome, the analysis with 125 cases and 250 controls would provide high power to detect an important association. The current paper and this supplemental material applies to the analysis of associations with CVD alone. At the time that the sampling was conducted there were a total of 127 subjects who had experienced a CVD event (the cases). A random sample of the complete cohort (the sub-cohort) of 350 subjects was selected. One the subjects did not have any remaining specimens and was not included in the study. Among the remaining 349 subjects, 29 were CVD cases leaving 320 CVD controls. Thus, the study sample comprised the 127 CVD cases and the 320 controls, 447 total. To determine the inverse sampling weights, the sampling fractions were employed within the 4 principal design strata defined by membership in the primary prevention versus secondary intervention cohort and assignment to the intensive versus conventional therapy.

2 Survey Sampling Analyses

Since a case-cohort design starts with a random sample of the full cohort, augmented by additional cases in the full cohort, the analysis must be conducted using weights inversely proportional to the sampling probabilities for cases and controls. These are called Hurwitz-Thompson estimators. Further, the expressions for the variance (standard error) of the estimators differ from those from simple random sampling. Thus, the analysis was conducted using sample survey methods that employ the inverse sampling weights. Doing so, the analysis provides estimates of the analysis results that would be expected had the full cohort been available for analysis.

3 Missing Data and Multiple Imputation

Unfortunately, there was a modest fraction of missing data. For the biomarker PON, the following table shows the numbers expected and missing at each point in time and the reason, either due to depletion or

loss-to-follow-up

	DCCT Baseline	DCCT Year 1	DCCT Closeout	EDIC Year 1/2	Total
Expected	447	446	436	430	1759
Missing n (%)	99 (22.1%)	45 (10.1%)	33 (7.6%)	84 (19.5%)	261 (14.8%)
Depletion n (%)	99 (22.1%)	45 (10.1%)	33 (7.6%)	78 (18.1%)	255 (14.5%)
Loss-to-follow-up n (%)	0 (0%)	0 (0%)	0 (0%)	6 (1.4%)	6 (0.3%)

The patterns missing for other biomarkers measured in plasma or in urine were similar.

All total, about 15% of the expected data is missing, allowing for CVD outcomes after which missing measures have no effect on the analysis. Virtually all of these are missing owing to depletion of material in preceding studies. Of these, the most troubling was the 22% missing the baseline value.

Accordingly the statistical technique of multiple imputation was employed to address the potential impact of these missing data (Rubin, 1987; van Buuren, 2012). Briefly, an imputation model is developed based on regression models for each variable as a function of the others, and the model is then applied to each missing value of each variable to impute a probable value for that instance based on the conditional expectation of the variable value plus a random error, the latter to preserve the variance and correlation structure of the data. For these data the imputation was conducted using the MICE procedure (Ragunathan et al. 2001) implemented in the R-function mice (White et al., 2011). The varables used included age, sex, duration of diabetes, DCCT primary versus secondary cohort, BMI, smoking, HDL, LDL, MBP, log(AER), and log(mean updated HbA1c) in all imputations (using the option "include"in function mice), and the CVD, retinopathy and renal status, as well as the four oxidative stress markers (all four on the log scale) based on their association with the variable being imputed (using option "minpuc" set to 0.2 in function mice).

Ten complete data sets (with no missing values) were generated and all analyses were then conducted separately using each data set. The results of the 10 analyses were then averaged using the method of Rubin and Schenker (1986).

4 Baseline Characteristics (Table 1)

Table 1 of the paper shows the weighted estimates (mean, proportion) of patient characteristics taking into account the inverse sampling probability weights.

Treatment Group	Cohort	CVD case/control	Weight
Intensive	Primary	Control	328/55
	Secondary	Control	331/119
Conventional	Primary	Control	345/55
	Secondary	Control	310/91
All	All	Case	127/127

This allows for the sampling probability of controls for each stratum defined by treatment group and cohort. Since all known CVD cases were included, the weight for all cases was 1. The sum of the weights in the first 4 strata (all controls), i.e. the sums of the numerators and denominators, equals 1314/340 and adding the 127 cases yields 1441/447.

The STATA **svy: mean** command provides an estimate of the population mean ($\widehat{\mu}$) and an estimate of the variance of $\widehat{\mu}$ $[\widehat{V}(\widehat{\mu})]$ accounting for the survey design used to collect the data as captured in the above weights. Note that for a binary characteristic (e.g. female vs male) the mean is in fact a proportion. In addition, however, we also desired an estimate of the underlying standard deviation of the observations. Since the variance of the estimate is σ^2/n, where σ^2 is the simple variance, then the estimate of the population standard deviation was simply $\sigma = n\widehat{V}(\widehat{\mu})$ that is reported using the "**estat sd**" command.

5 Longitudinal Biomarker Models (Table 2)

Table 2 presents the longitudinal analysis of each biomarker as the dependent variable. Since patients entered the study with an HbA1c of about 9% and the intensive group lowered that to 7% during the first year, one question is whether there was a significant change in the biomarker from year 1 to 2 and whether this change differed between groups. Then at DCCT closeout subjects were referred to their own healthcare providers and during during the first 1-2 years the HbA1c dropped from about 9% to 8% in the conventional group and increased from about 7% to 8% in the intensive group. Thus another question was whether there was a significant change in the biomarker from year 3 to 4 and whether this change differed between groups

This analysis was conducted using the SAS procedure SURVEYREG in which the patient was a cluster containing the measures at the 4 time points. The model then contained an effect (class) for group (intensive/conventinal), time (1, 2, 3 or 4) and their interaction. The model used the weights as shown for Table 1.

The estimate function was then used to compute contrasts among the model coefficients to estimate specific effects such as the change in the biomarker from year 1 to 2 in each group (separately) and the difference between groups. The estimate statement contrast coefficients were derived using the methods described in Littell, Freund and Spector (1991).

6 Cox Proportional Hazards Models (Table 3)

Table 3 of the paper then presents prospective Cox Proportional Hazards (PH) models of the association of each biomarker with the risk of CVD. These models used the biomarker values at times 1 - 4 as a time dependent covariate. The models used the method of Barlow (1994) as implemented by Therneau and Li (1999). The models used "start-stop" notation to indicate the day (since randomization) that the biomarker value was updated. The models used an offset equal to the log of the weights. The Lin-Wei (1989) robust estimate of the covariance matrix of the model coefficients was employed to compute confidence limits and p-values.

The case-cohort design was originally described by Prentice (1986) who provided a generalization of the original PH model to conduct the analysis. Then Barlow (1994) described a simpler method of analysis that could be implemented using available software such as the SAS PROC PHREG.

Let u_i be an indicator variable to denote membership in the subcohort, = 1 if yes, 0 otherwise; δ_i.denote whether the subject had an event at time t_i or is right censored at time t_i. Then the Barlow (1994) analysis is based on the likelihood

$$\widetilde{L}(\beta) = \prod_{i=1}^{n_{CC}} \left[\frac{e^{\mathbf{x}_i'\beta}}{(1-u_i)e^{\mathbf{x}_i'\beta} + \frac{1}{\alpha} \sum_{\ell \in S(t_i)} e^{\mathbf{x}_\ell'\beta}} \right]^{\delta_i}, \tag{1}$$

where n_{CC} is the size of the case-cohort of cases and controls, n is the size of the sub-cohort, and $\alpha = n/N$ so that the contribution of the subcohort is upweighted by N/n, the inverse sampling weight. In EDIC the sample weights were computed separately within strata defined by treatment group (intensive versus conventional) and study cohort (primary prevention versus secondary intervention), 4 strata in total. The weights were

Treatment Group	Cohort	Weight
Intensive	Primary	348/58
	Secondary	363/125
Conventional	Primary	378/58
	Secondary	352/108

Note that the weights sum to 1441/349. One subject from the original sub-cohort of 350 randomly chosen subjects did not have any specimens remaining for assay. This subject was not employed in any analyses herein, so the sub-cohort sample size enters as 349 rather than 350 as originally selected.

The original Barlow model was fit using "extra" observations in the data set using counting process structure. This can be generalized to the case where there are separate sampling fractions for cases and

controls. It is best described by example. Consider different subjects

i	u_i	δ_i	start	stop
1	1	0	0	t_1
2	1	1	0	t_2
3	0	1	t_3^-	t_3

Subject 1 is a sub-cohort control at risk for the entire time to censoring (t_1). Subject 2 is a sub-cohort case that is at risk up too the event time (t_2). Subject 3 is an extra case that was not in the subcohort. That subject is at risk only at the precise event time for that case, where $t^- = t - \epsilon$ for some small ϵ. So all cases have a record at risk for the instant of the event, and sub-cohort cases also are at risk up to the moment prior to the event.

With time dependent covariates, subjects 1 and 2 would have records documenting the start and stop times at which the covariate process changes over time. However, for a non-sub-cohort case, there would be only one record with (start, stop) times of (t_3^-, t_3) and where the value of the time-dependent covariate is the last recorded value.

7 Software

The multiple imputations were performed in R 3.0.0. The analyses for Table 1 were conducted using the SAS PROC SURVEYMEANS and Stata using the svy: mean command. The analyses in Table were performed using the SAS PROC SURVEYREG and also using SURVEYMEANS. The analyses in Tables 3 and 4 were generated using SAS PROC PHREG. SAS version 9.2 or 9.4 was employed, and Stata version 15

8 References

Barlow WE. Robust variance estimation for the case-cohort design. *Biometrics* 50(4):1064-72, 1994.

Kalbfleisch JD, Prentice RL. *The Statistical Analysis of Failure Time Data.* New York: John Wiley & Sons, 1980.

Lin DY, Wei LJ. The robust inference for the Cox proportional hazards model. *Journal of the American Statistical Association.* 84: 1074–1078, 1989.

Littell RC, Freund RJ and Spector PC. *SAS® System for Linear Models.* Third Edition, Cary, NC: SAS Institute, Inc. 1991.

Raghunathan TE LJ, Hoewyk JV, Solenberger P. A multivariate technique for multiply imputing missing values using a sequence of regression models. *Surv Methodol* 27:85-95, 2001.

Rubin DB. *Multiple Imputation for Nonresponse in Surveys.* New York: John Wiley & Sons, 1987.

Rubin DB, Schenker N. Multiple imputation for interval estimation from simple random samples with ignorable nonresponse. *Journal of the American Statistical Association* 81(394):366-374, 1986;

Therneau TM, Li H. Computing the Cox model for case cohort designs. *Lifetime Data Anal* 5(2):99-112, 1999.

van Buuren S. *Flexible Imputation of Missing Data*, Chapman & Hall/CRC, 2012.

White IR, Royston P, Wood AM. Multiple imputation using chained equations: issues and guidance for practice. *Stat Med.* 30(4):377-399, 2011.
